# Functionalized Graphene Derivatives and TiO_2_ for High Visible Light Photodegradation of Azo Dyes

**DOI:** 10.3390/nano10061106

**Published:** 2020-06-03

**Authors:** Álvaro Pérez-Molina, Sergio Morales-Torres, Francisco J. Maldonado-Hódar, Luisa M. Pastrana-Martínez

**Affiliations:** Carbon Materials Research Group, Department of Inorganic Chemistry, Faculty of Sciences, University of Granada, Avda. Fuente Nueva, s/n ES18071 Granada, Spain; alpemo@correo.ugr.es (Á.P.-M.); semoto@ugr.es (S.M.-T.); fjmaldon@ugr.es (F.J.M.-H.)

**Keywords:** graphene oxide, TiO_2_, heteroatom doping, photocatalysis, Orange G, scavengers

## Abstract

Functionalized graphene derivatives including graphene oxide (GO), reduced graphene oxide (rGO), and heteroatom (nitrogen/sulphur (N/S) or boron (B))-doped graphene were used to synthesize composites with TiO_2_ (T). The photocatalytic performance of composites was assessed for the degradation of Orange G dye (OG) under simulated solar light. All the prepared graphene derivatives—TiO_2_ composites showed better photocatalytic performance than bare TiO_2_. A higher photocatalytic activity was found for the composites containing GO and N/S co-doped rGO (*k_app_* = 109.2 × 10^−3^ and 48.4 × 10^−3^ min^−1^, for GO-T and rGONS-T, respectively). The influence of both initial solution pH and the reactive species involved in the OG degradation pathway were studied. The photocatalytic activity of the samples decreased with the increase of the initial pH (from 3.0 to 10.0) due to the occurrence of electrostatic repulsive forces between the photocatalysts surface and the molecules of OG, both negatively charged. The use of selective scavengers showed that although the photogenerated holes dominate the degradation mechanism, radicals and singlet oxygen also participate in the OG degradation pathway. In addition, reutilization experiments indicated that the samples were stable under the reaction conditions used.

## 1. Introduction

The growing industry and population is leading to a decrease of water resources quality. Up to date, several contaminants have been detected in water and wastewater such as nitrates, phosphates, metals, dyes, pesticides, pharmaceuticals, personal care products, endocrine disruptors, etc. Organic dyes are pollutants especially difficult to remove because of their high solubility and stability [1]. Advanced oxidation processes (AOPs) are considered efficient treatments for water polluted with recalcitrant and non-biodegradable compounds [2]. Heterogenous photocatalysis is a very attractive option because it is possible to use sunlight, the availability of non-poison materials, and low cost. In addition, it has demonstrated a high efficiency in degrading a wide range of water pollutants [3,4,5]. Among the various semiconductors used as photocatalysts such as TiO_2_, ZnO, ZrO_2_, CdS, ZnS, WO_3_ etc., TiO_2_ has been typically studied because of its high efficiency and stability for the degradation of inorganic and organic pollutants. However, its application in the visible range of the solar spectrum is limited by the low quantum yield and the wide band gap of TiO_2_ (i.e., 3.2 eV for anatase) [6,7,8,9]. Several strategies have been developed to expand the response of TiO_2_ into the visible range spectra namely, the addition of electron donors [10], noble metals [11], metal ion or anion doping [12,13], dye sensitization [14], synthesis of TiO_2_ with exposed {001} facets [15], heterojunction with different types of active materials [16,17], combination with carbon materials [18], etc.

Carbon materials such as graphene (a two-dimensional allotrope of carbon) and its well-known derivatives including graphene oxide (GO) and reduced graphene oxide (rGO) have demonstrated the ability to enhance the photocatalytic performance of TiO_2_ by producing a synergistic effect between both phases [18,19]. In fact, the delocalized conjugated system of π bonds in graphene materials could accept photogenerated electrons, avoiding the electron-hole recombination. Furthermore, the presence of oxygenated functionalities on GO (and in less extent in the case of rGO) could provide reactive sites to facilitate the assembly of the semiconductors and graphene sheets [19].

Different methods of synthesis have been used for the preparation of graphene based-TiO_2_ composites for photocatalytic applications including the degradation of organic pollutants, production of H_2_, reduction of CO_2_, as well as supercapacitors, among others, under visible illumination. Liang et al. [20] reported the synthesis of rGO-TiO_2_ composites by a one-step hydrothermal method and using ethanol/water as a reducing agent, with complete dye (Rhodamine B) degradation being achieved under UV–Vis irradiation for 30 min. Zhao et al. [21] used a two-step method (based on the hydrolysis and vacuum furnace at 700 °C) for the preparation of graphene-TiO_2_ composites to remove 70% of methylene blue under visible light irradiation (λ = 450 nm) for 200 min. Other authors [22] reported Pt/GO-TiO_2_ composites by the hydrothermal method with TiO_2_ nanocrystals with exposed {001} and {101} facets for selective CO_2_ conversion to CH_4_, resulting in the selectivity of CO_2_ conversion to the CH_4_ product closely to 100% under simulated solar irradiation. Fattahi et al. [23] prepared graphene-TiO_2_ composites by simultaneous hydrothermal synthesis and GO reduction. The photocatalytic performance of these composites for the formation of 2-hydroxyterephthalic acid (HTPA) under visible light (λ = 405 nm) was optimized by changing different synthesis parameters, such as stirring time and speed, the load of TiO_2_ and GO, reaction time and ethanol/water ratio. They obtained an increase of the HTPA formation rate for the optimized graphene-TiO_2_ composite in comparison with bare TiO_2_. Police et al. [24] reported the synthesis of rGO-TiO_2_ nanotube composites by the hydrothermal method showing a remarkable photocatalytic activity towards H_2_ production (12.9 times higher than commercial TiO_2_ under natural sunlight), as well as in other applications such as supercapacitors. Recently, Ashraf et al. [25] reported the synthesis of graphene/TiO_2_/Ag composites by the combination of a sonochemical method and freeze-drying with a high visible light photocatalytic activity for the degradation of an azo dye (C.I. Reactive Yellow 2). They obtained 100% of dye degradation after 90 min under visible light. 

Nevertheless, the optimization of the electronic properties of graphene is a critical topic today and growing interest has been devoted to the use of heteroatom-doped graphene (rather than the addition of noble metals) to produce high-performance photocatalytic composites under visible light. Chemical doping with heteroatoms such as oxygen (O) [26], nitrogen (N) [27,28,29], boron (B) [30], phosphorus (P) [31], or sulfur (S) [32,33], etc., can improve the photocatalytic performance of graphene materials by tailoring its electronic properties [34] and increasing their reactive catalytic sites to be used such as catalysts supports or even as a photocatalyst on their own [35]. 

Mou et al. [36] fabricated a composite based on N-doped rGO-TiO_2_ through a solvothermal treatment showing higher photocatalytic activity for H_2_ production in comparison with the composite prepared with rGO. Pedrosa et al. [37] synthetized N- and S-doped rGO-TiO_2_ composites for the degradation of a pharmaceutical compound observing that the oxygen content in the precursor material plays an important role in the catalytic performance. Another study reported [38] the degradation of ca. 95% and 70% of methylene blue and 50% and 65% of rhodamine B in 60 min using B- or N-doped graphene-TiO_2_ composites, respectively. The photocatalytic activity seems to be affected by the electronic structure of graphene. 

However, the design of composites using graphene with an optimized concentration of external atoms and defects still remains a challenge in photocatalytic applications and needs to be more exhaustively studied [34,37,39,40]. 

In the present work, GO was co-doped with different heteroatoms, in particular N/S or B, following a hydrothermal process. Graphene derivatives were used to synthesize composites with TiO_2_ by the liquid phase deposition method. The synthetized materials were deeply characterized in order to identify the effect of different heteroatoms on their physicochemical properties and photocatalytic performance. The photodegradation of Acid Orange G (OG) dye under simulated solar radiation was studied as a reaction model for the catalysts screening. The selected azo dye is typically used in the paper and textile industry showing carcinogenic and mutagenic activity [41,42,43,44]. The optimization of experimental conditions (i.e., pH), the identification of photoactive species, as well as the stability of materials was also evaluated. 

## 2. Materials and Methods 

### 2.1. Synthesis of Graphene Oxide (GO)

GO was synthetized from graphite (powder < 20 μm) following the modified Hummers method [45]. Briefly, 240 mL of sulfuric acid, H_2_SO_4_ (96–99%, supplied by PanReac AppliChem, Darmstadt, Germany) was added slowly on both 5 g of graphite (Sigma-Aldrich, St. Louis, MO, USA) and 5 g of sodium nitrate, NaNO_3_ (99%, Acros Organics, Geel, Belgium, keeping it under agitation and controlling the temperature with an ice bath. Then, 30 g of potassium permanganate, KMnO_4_ (99%, PanReac AppliChem, Darmstadt, Germany) was added slowly under stirring to the above solution and heated at 35 °C for 10 h. Afterwards, 1000 mL of water and 30 mL of hydrogen peroxide, H_2_O_2_ (30% w/w, PanReac AppliChem, Darmstadt, Germany) were added very slowly to the suspension and after 30 min, the material was filtered. The obtained paste was washed repeatedly with water until it reaches pH of 5.5–6.0. The graphite oxide material obtained was dispersed in water (1.0 g in 500 mL) and sonicated for 1 h.

### 2.2. Synthesis of Heteroatom Reduced Graphene Oxide (rGO) 

N/S- and B-doped graphene were prepared with a GO suspension (1.0 g L^−1^) and thiourea, CH_4_N_2_S (99%, Alfa Aesar, Haverhill, MA, USA) [46], or boric acid, H_3_BO_3_ (Acros Organics, Geel, Belgium) [40], as N/S or B precursors, respectively by hydrothermal reduction [47]. The GO: Precursor ratio was selected as 1:10, in agreement with the optimized value in a previous study [46]. In a typical synthesis, an appropriate amount of thiourea or H_3_BO_3_ was dissolved into 60 mL of GO suspension and stirred for 10 min followed by sonication for 15 min. The above mixture was placed into a 100 mL Teflon vessel and sealed in a stainless-steel autoclave (Parr Instruments, Moline, IL, USA, Mod. 4748) to perform a hydrothermal treatment in an oven at 180 °C for 12 h. The resultant materials were washed with distilled water and exchanged with *tert*-butanol for 48 h. Finally, the freeze-drying process was used to remove the solvent (20 h). The materials were labelled as rGONS or rGOB when using thiourea or boric acid as precursors, respectively. The rGO material was also synthetized with comparative purposes, following the same procedure but without the addition of thiourea or boric acid. 

### 2.3. Synthesis of Graphene Derivative-TiO_2_ Composites

The synthesis of the photocatalytic composites was carried out by the liquid phase deposition method (LPD), as previously reported [6]. Briefly, the precursors, ammonium hexafluorotitanate (IV), (NH_4_)_2_TiF_6_ (99%, Sigma-Aldrich, St. Louis, MO, USA), and H_3_BO_3_ (0.1 and 0.3 mol L^−1^, respectively) were added to a 100 mg L^−1^ suspension of GO, rGO, rGONS, or rGOB. The carbon loading was maintained at ~3.5 wt%. The mixture was heated in a silicon bath at 60 °C for 5 h under dynamic stirring. The precipitate was filtrated under a vacuum, washed repeatedly with distilled water, and dried at 80 °C for 8 h. Finally, the obtained solid was treated under N_2_ flow in an oven at 200 °C, 5 °C min^−1^ for 2 h. Bare TiO_2_ (referred as TiO_2_) was also synthetized following the same methodology, without the addition of any graphene derivative. The TiO_2_ composites prepared with GO, rGO, rGONS, and rGOB are denoted, as GO-T, rGO-T, rGONS-T, and rGOB-T, respectively. 

### 2.4. Characterization Techniques

A thermogravimetric (TG) analysis of the composites was determined by heating from 40 to 950 °C (air flow) at 20 °C min^−1^ using a SHIMADZU TGA-50H thermobalance. The NICOLET 510P spectrometer with an attenuated total reflection accessory and a ZeSn as ATR crystal was used for the determination of ATR-IR spectra. An elemental CHNS-O Analyzer Flask (1112 Series) from Thermo Finigan was used to obtain the total oxygen content of samples. The N_2_ adsorption-desorption isotherms at −196 °C were carried out using a Quantachrome Quadrasorb SI equipment. The Brunauer–Emmett–Teller (BET) equation was applied to calculate the apparent surface area (S_BET_) [48,49]. Pore size distributions and the mean pore diameter (d_pore_) were determined by using the quenched solid density functional theory (QSDFT) as reported elsewhere [19]. The total pore volume (V_total_) was obtained considering the volume of N_2_ adsorbed at P/P_0_ = 0.95 [50]. The point zero of charge (pH_PZC_) of the materials was determined following the method described elsewhere [51,52]. The LEO (Carl Zeiss) GEMINI-1430-VP microscope was used to analyze the morphology of the materials by scanning electron microscopy (SEM). The X-ray diffractograms were obtained in a Philips PW 1710, using the CuKα radiation and a nickel filter that removes the κβ radiation. The average crystal size of the samples was determined using the Scherrer equation. The UV–Vis spectrophotometer (CARY 5E from VARIAN) equipped with a diffuse reflectance accessory (DRA) was used for the analysis of the optical properties of photocatalysts. The band gap of the materials was calculated from the corresponding Tauc plots using (Abs·hν)^1/2^ units as a function of energy (eV). 

### 2.5. Photocatalytic Experiments

The photocatalytic degradation of Acid Orange G (OG) dye (Sigma-Aldrich, St. Louis, MO, USA) was evaluated under simulated solar light at ca. 28 °C. In a typical experiment, a Pyrex reactor was filled with 50 mL of OG aqueous solution (20 mg L^−1^ or 4.42 × 10^−5^ mol L^−1^). During the photocatalytic experiment, the aqueous solution was stirred with a magnet and an oxygen flow was used. The concentration of the photocatalyst was 1.0 g L^−1^.

The experiments were performed with a 500 W m^−2^ of irradiance power using a SOLAR BOX 1500 e (CO.FE.MEGRA, Milano, Italy) with a 1500 W Xenon lamp. In order to establish the adsorption-desorption equilibrium, the suspensions were maintained in absence of light during 60 min. Samples were withdrawn from the reaction mixture and filtered with polyether sulfone syringe filters (0.45 µm pore size). Photolysis experiments (in the absence of catalyst) were also performed. Samples were analyzed using a UV-spectrophotometer model UV-1800 Shimadzu. For all the photocatalytic experiments, the absorption spectra (λ_máx_ = 485 nm) of OG were measured at different reaction times. The total organic carbon (TOC) analysis was performed in a Shimadzu TOC-5000A apparatus. The experiments were performed at different pH values, i.e., 3.0, 6.0 (natural), and 10.0, by adding HCl 0.1 or NaOH 0.1 M, respectively. The photocatalytic degradation pathway of OG was studied using an ethylenediaminetetraacetic acid (EDTA, 1.0 mM), furfuryl alcohol (FFA, 1.0 mM), *tert*-butanol (*t*-BuOH, 1.0 mM), and as a hole, singlet oxygen (^1^O_2_) and radical scavengers, respectively [6].

The photocatalytic degradation can be described by the following equation:

(1) $$\left[OG\right]={\left[OG\right]}_{0}\;\times \;e^{-k_{app}\times t}$$

where *k_app_* is the pseudo-first order kinetic constant, *t* is the reaction time, and [OG]_0_ and [OG] denote the pollutant concentration at *t* = 0 and *t* = t, respectively. The values of *k_app_* were obtained by a non-linear regression.

## 3. Results and Discussion

### 3.1. Materials Characterization

The graphene content (wt%) in all the prepared composites was analyzed by TG (not shown) calculated from the weight loss of the respective graphene derivative-TiO_2_ composites by burning the carbon phase during the TG experiments under air flow. The results corroborated at ca. 4.0 wt% carbon (in agreement with the nominal carbon content, i.e., ~3.5%). This content was chosen in accordance with the best photocatalytic activity of the composites prepared with TiO_2_ and GO, as reported elsewhere [6,19]. 

**Figure 1:**
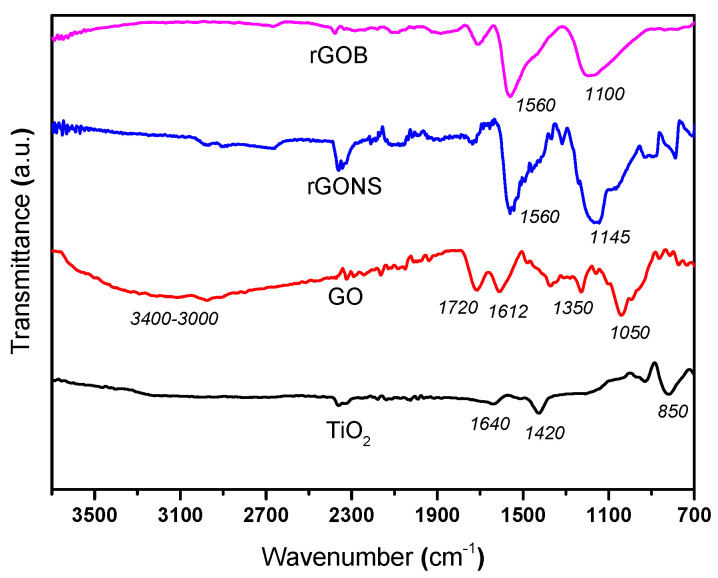
FT-IR spectra of TiO_2_ (T), graphene oxide (GO), reduced graphene oxide nitrogen/sulphur (rGONS), and reduced graphene oxide boron (rGOB).

The ATR-IR spectra of bare TiO_2_, GO, and doped graphene samples are displayed in Figure 1. The TiO_2_ spectrum shows a band at ca. 850 cm^−1^ associated to the Ti-O vibration [53]. The presence of a band at around 1640 cm^−1^ is related to the presence of the Ti-OH group as well as the bending vibration of coordinated water [50,53], while the TiO_2_ lattice vibrations are assigned to the peak at ca. 1420 cm^−1^ [54,55]. 

For the GO spectrum, different bands were observed related to the presence of oxygen functionalities. The bands at ca. 1050 and 1350 cm^−1^ are assigned to the stretching vibration of C-O and to the stretching of C-OH groups, respectively while the bands at ca. 1612, 1720, and around 3000–3400 cm^−1^ are attributed to the skeletal vibration of graphene sheets, to carbonyl groups (C=O), and to the vibrations of C-OH groups, respectively [19]. 

Regarding the rGONS sample, the FT-IR spectrum shows two main peaks, one of them associated to the vibration of sp^2^ aromatic C=C and C=N bonds at ca. 1560 cm^−1^ and another attributed to the stretching vibration of the C-S-C groups at ca. 1100–1145 cm^−1^ [37,46,56,57,58]. In the case of rGOB, there are also two main peaks in the spectrum, attributed to the vibration of C=C bond (at ca. 1560 cm^−1^) and to the vibration of B-C bond and C-O bond (at ca. 1100 cm^−1^), as previously observed [46,59]. On the other hand, the peaks associated to the oxygen functional groups in the doped-graphene materials exhibited lower intensities in comparison with the obtained for the GO sample (Figure 1). These results could be due to the partial removal of these groups after hydrothermal treatment. Thus, the elemental analysis of the graphene-based materials indicated a percentage of oxygen content (wt%) of 53.5%, 23.2%, and 21.4%, for GO, rGO, and rGONS, respectively (results not shown). These results suggested a considerable deoxygenation as well as heteroatom incorporation (ca. 1.8% and 1.3%, for N and S, respectively). 

The XPS analysis of the rGONS sample (labelled as rGO-NS-10) and B-doped graphene (prepared by a similar synthesis procedure to the rGOB sample) was obtained and discussed in previous studies [40,46]. The nitrogen species in the rGONS material were the amino group (-NH_2_), N-pyridinic species (N6), N-pyrrolic form (N5), and N-graphitic (NQ), whereas the sulphur groups correspond mainly to S-thiophene. Regarding the rGOB sample, the most intense peak corresponds to boron replacing carbon in the hexagonal lattice (i.e., BC_3_).

**Figure 2:**
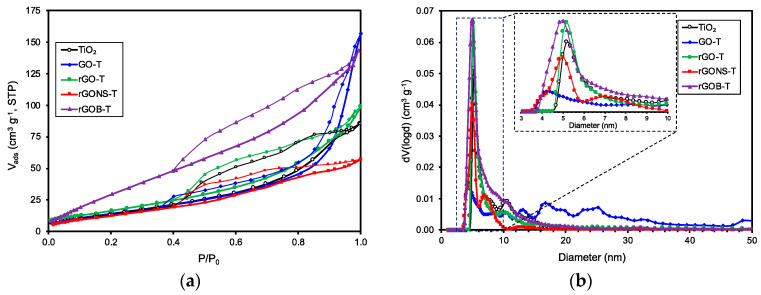
(**a**) N_2_ adsorption-desorption isotherms and (**b**) pore size distributions obtained by the quenched solid density functional theory (QSDFT) method of TiO_2_, GO-T, rGO-T, rGONS-T, and rGOB-T.

Physical adsorption of N_2_ at −196 °C was carried out to determine the textural properties of the materials. Table 1 summarizes the apparent surface area (*S_BET_*), total pore volume (*V_pore_*), and the mean pore diameter (*d_pore_*) of the materials. N_2_ adsorption-desorption isotherms for TiO_2_ and the prepared graphene derivative—TiO_2_ composites are shown in Figure 2a. The N_2_ isotherms of the samples showed an adsorptive behaviour of type-IV (in agreement with the IUPAC classification) [48], attributed to mesoporous materials. The larger volume of adsorbed N_2_ at high relative pressures can be related to capillary condensation in mesopores. Moreover, the presence of a hysteresis loop of type H3 in the samples could be due to the adsorbents with slit-shaped pores or the presence of aggregates formed by plate particles as reported elsewhere [6,50].

In general, TiO_2_ and graphene based-TiO_2_ composites exhibited surface areas, S_BET_, of ca. 40–80 m^2^ g^−1^, rGOB-T being the sample with the highest surface area (80 m^2^ g^−1^). In general, all the prepared composites with graphene derivatives presented a higher total pore volume than the TiO_2_ sample. The pore size distribution (PSD) of the samples are depicted in Figure 2b. The results indicated mean pore sizes between 5.0 and 10.3 nm, with the exception of GO-T that presented a wider PSD with larger mesopores, i.e., 16.7 and 25.2 nm. This fact could be associated to the high amount of oxygen functionalities, which facilitate the separation of graphene layers and coating of TiO_2_ particles during the synthesis of composite preparation. 

The pH_pzc_ values obtained for both TiO_2_ and composite materials presented an acidic character, i.e., pH_pzc_ = 3.0–3.5 (Table 1), which should be explained with TiO_2_ precursors, the low temperature treatment (200 °C), and the presence of acidic groups on graphene surfaces. 

**Figure 3:**
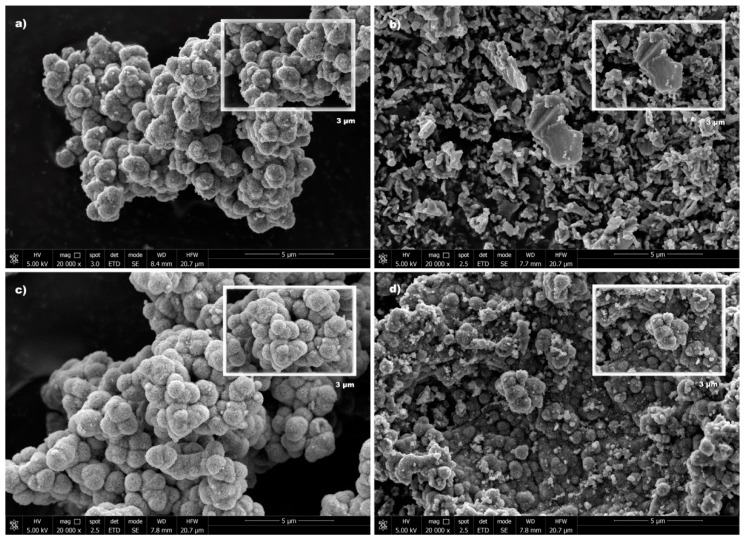
SEM micrographs of (**a**) TiO_2_, (**b**) GO-T, (**c**) rGONS-T, and (**d**) rGOB-T.

Figure 3 shows representative SEM micrographs of TiO_2_ and graphene-based composites. The morphology of TiO_2_ (Figure 3a) shows spherical particles aggregated with each other forming a cluster of TiO_2_ particles. 

The SEM micrograph of the GO-T composite (Figure 3b) shows a well distribution of TiO_2_ particles around the GO sheets resulting in the formation of a kind of platelets uniformly covered by TiO_2_ nanoparticles [6]. Different morphologies were obtained for the heteroatom doped-graphene-TiO_2_ composites (Figure 3c,d) in comparison with the morphology observed for GO-T, since the presence of well-separated platelets was not so notorious. The surface morphology of rGONS-T and rGOB-T seems to be similar to that of bare TiO_2_ presenting larger or smaller clusters of particles, respectively. 

**Figure 4:**
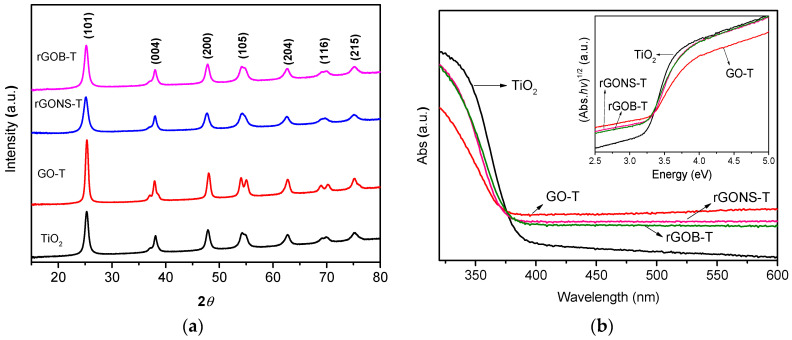
(**a**) XRD patterns of TiO_2_, GO-T, rGONS-T, and rGOB-T, (**b**) UV–Vis spectra and Tauc’s plots versus the energy in eV of TiO_2_ and graphene derivative-TiO_2_ composites (inset).

The XRD patterns of the TiO_2_, GO-T, rGONS-T, and rGOB-T samples are depicted in Figure 4a. TiO_2_ anatase particles were detected in all the prepared materials. The major diffraction peaks at 2θ values of 25.2, 37.7, 48.1, and 53.5° were associated to the lattice planes of (101), (004), (200), and (105), respectively [60]. No significant diffraction peaks of carbon were observed in the XRD patterns of GO-T, rGONS-T, and rGOB-T composites. These results could be associated to the low amount of carbon material present in the composites (i.e., 3%–4%). Particles sizes of 9.7, 12.1, 7.6, and 8.9 nm were calculated for TiO_2_, GO-T, rGONS-T, and rGOB-T photocatalysts, respectively (Table 1) displaying the doped-graphene composites with N/S or B, lower particles sizes in comparison with both GO-T and TiO_2_.

The UV–Vis absorption spectra of TiO_2_ and graphene derivative-TiO_2_ composites are depicted in Figure 4b. For all the samples, a strong band at ca. <400 nm corresponding to the intrinsic bandgap transition of TiO_2_ was observed. Furthermore, all the graphene-TiO_2_ composites (i.e., GO-T, rGONS-T, and rGOB-T) display a decrease in wavelength on the TiO_2_ absorption band. These results can be associated to the electronic interaction between semiconductor and carbon as well as to the occurrence of defect in the structure of TiO_2_ [61]. Moreover, it is noteworthy that for all the graphene-TiO_2_ materials an increase of the absorption range at a wavelength higher than 380 nm (visible range) in comparison with TiO_2_ was observed. This effect is normally associated to the intrinsic light absorption capacity of graphene as a carbon material and also to the electronic transitions between both phases [37]. The inset of Figure 4b exhibits the Tauc’s plots versus the energy (eV). The calculated E_g_ of TiO_2_, GO-T, rGONS-T, and rGOB-T were 3.20, 2.98, 3.12, and 3.12 eV, respectively (Table 1). The lowest E_g_ of GO-T and the graphene-doped composites in comparison with bare TiO_2_ may be associated to the presence of Ti-O-C bonds between carbon materials and TiO_2_ [37].

### 3.2. Photocatalytic Degradation of OG

**Figure 5:**
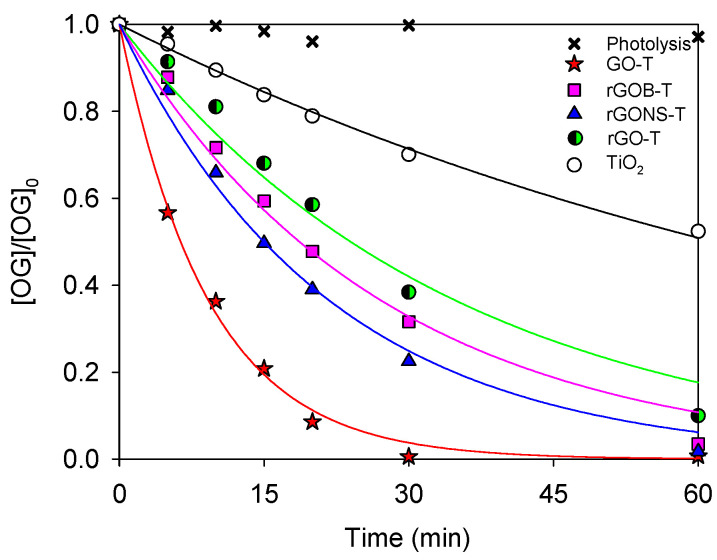
Normalized concentration of OG (Orange G dye) as a function of time in photolysis and photocatalysis (TiO_2_, rGO-T, GO-T, rGONS-T, and rGOB-T).

The photocatalytic performance of TiO_2_ and graphene derivatives-TiO_2_ composites for OG degradation (at natural pH, 6.0) under simulated solar light is shown in Figure 5. The OG conversion after 60 min (X_OG_), pseudo-first order kinetic rate constant (*k_app_*), and regression coefficient (r^2^) are summarized in Table 2. The photolysis experiment was carried out in the absence of a photocatalyst and under simulated solar light. Under these conditions, the degradation of OG was approximately 6.0% after 60 min, indicating that the pollutant is very light-stable under non-catalytic conditions. On the other hand, dark phase experiments (in the absence of light) were also performed to evaluate the adsorption capacity of the materials (not shown). The results obtained were 6.0%, 10.0%, 8.0%, 8.0%, and 7.0% for TiO_2_, GO-T, rGO-T, rGONS-T, and rGOB-T, respectively, detecting that the adsorption equilibrium was reached after 60 min for all the photocatalysts. Once the adsorption-desorption equilibrium was achieved, OG degradation experiments were carried out under simulated solar light. 

The presence of graphene derivatives, such as GO, rGO, rGONS, and rGOB, increased the efficiency for the OG degradation in comparison with TiO_2_ (OG conversion of 99.8%, 90.0%, 98.2%, 96.5%, and 47.6% for GO-T, rGO-T, rGONS-T, rGOB-T, and TiO_2_ respectively, Figure 5 and Table 2). These results suggest a synergistic effect between graphene materials and TiO_2_, although this effect depends on the type of graphene derivative used in the composite. In particular, the composite prepared with GO (i.e., GO-T) showed the best performance for the OG degradation under simulated solar light (k_app_ = 109.2 × 10^−3^). The results of TOC removal were found to follow the same trend as a photocatalytic conversion, i.e., GO-T, rGONS-T, rGOB-T, rGO, and TiO_2_ produced respectively 40%, 22%, 17%, 17%, and 15% of TOC reduction after 60 min of solar irradiation. 

The lower photocatalytic activity obtained for rGO-T, rGONS-T, and rGOB-T when compared with the GO-T composite could be attributed to the lower amount of oxygen functionalities, resulting in a weaker interaction between graphene derivatives and TiO_2_. The lowest band gap energy of the GO-T composite (Table 1) as well as the pronounced quenching of photoluminescence in the GO-T composite [6], suggest that GO can effectively accept the photoexcited electrons to hinder electron-hole recombination. These effects combined with the good assembly and interfacial coupling between the TiO_2_ and the GO sheets, as observed by SEM images (Figure 3b), may promote charge migration between both phases after photoexcitation, leading to an increase in the efficiency of the photocatalytic process for the GO-T composite.

Nevertheless, the doped graphene derivative-TiO_2_ composites also showed better performance when compared with both rGO-T and bare TiO_2_. These results can be explained due to the presence of N/S or B-doping, which favoured a lower band-gap energy (E_g_) for both rGONS-T and rGOB-T in comparison with rGO-T and TiO_2_ materials (3.12, 3.12, 3.15, and 3.20 eV, respectively, Table 1). The photocatalytic activity can be also related with the enhancement of electronic conductivity as well as the recovery of the sp^2^ graphene network and the decrease of defects within the plane associated with heteroatom incorporation [34,36,62]. In general, the results suggest that the addition of any graphene derivative in the TiO_2_ matrix can promote the photocatalytic activity of TiO_2_ under solar light due to the charge transfer of photo-generated electrons between TiO_2_ and graphene that can limit the electron-hole recombination, permitting the graphene-TiO_2_ composites to produce a higher amount of radicals under solar radiation [63]. Both GO-T and rGONS-T materials were selected to study the effect of initial pH on the OG conversion, the photoactive species involved in the reaction, and reusability cycles.

#### 3.2.1. Influence of pH on OG Degradation

**Figure 6:**
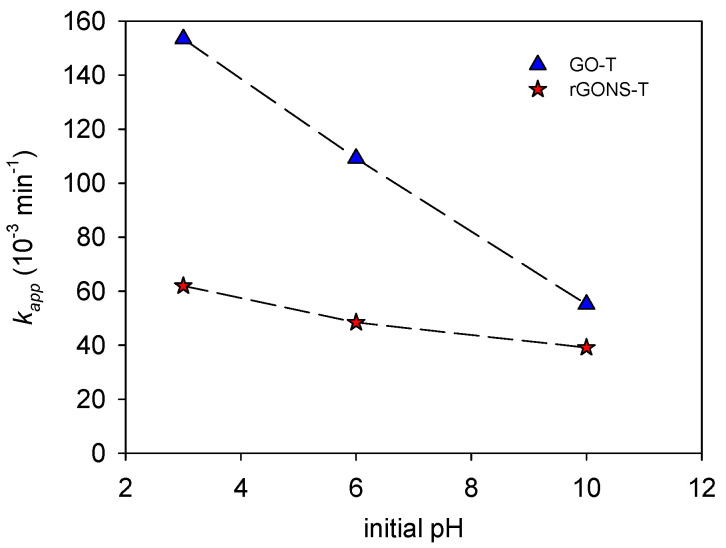
Pseudo-first order kinetic rate constant (*k_app_*) for different initial solution pH over the GO-T and rGONS-T composites.

The photocatalytic degradation of OG at initial pH values of 3.0, 6.0 (natural pH), and 10.0 is shown in Figure S1a,b of the Supplementary Materials for both GO-T and rGONS-T, respectively. The obtained *k_app_* constants for the different pH values are illustrated in Figure 6 while the OG conversion (*X_OG_*), rate constant (*k_app_*), and regression coefficient (*r^2^*) at different pH values are summarized in Table 2.

It can be observed that the photocatalytic performance of photocatalysts decreased as the initial pH value increased, as observed for the reaction rate constants for both materials when the initial pH increased from pH 3.0 to 10.0 (*k_app_* = 153.4 × 10^−3^ and 62.0 × 10^−3^ min^−1^ for GO-T and rGONS-T, respectively for pH 3.0 and *k_app_* = 55.1 × 10^−3^ and 39.1 × 10^−3^ min^−1^ for GO-T and rGONS-T, respectively for pH 10.0, Figure 6).

It is well known that the initial pH value can modify the surface charge density of catalyst and the ionization state of organic molecules at the same time, among others [64]. For pH values higher than the pH_PZC_ of TiO_2_, the surface charge becomes negative (TiO^−^), while for pH values lower than the pH_PZC_, the surface charge is positive (TiOH_2_^+^). OG is a molecule with a negative charge (OG^−^) in the solution due to the deprotonation of the sulfonic group ^−^SO_3_, (pK_a_ = 1.0) [65]. When the initial pH is 3.0, the surface of both GO-T (pH_PZC_ ≈ 3.1) and rGONS-T (pH_PZC_ ≈ 3.3) is positively charged, while OG is negatively charged. Thus, electrostatic attraction forces are expected. On the contrary, at higher pH values (i.e., natural pH of 6.0 and 10.0), a lower photodegradation rate for both materials was observed due to the occurrence of electrostatic repulsive forces between the negatively charged catalysts surface and the OG^−^ species.

#### 3.2.2. Photocatalytic Degradation Pathway

**Figure 7:**
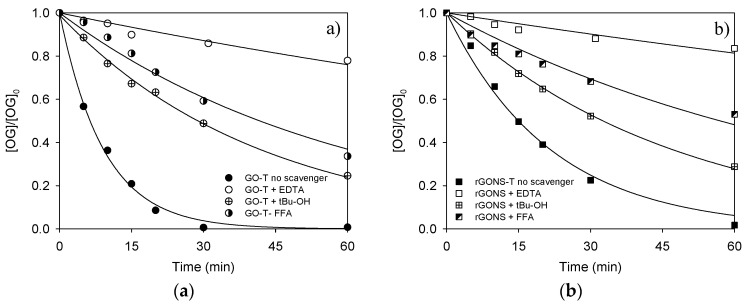
Normalized concentration of OG as a function of time with and without scavengers for (**a**) GO-T and (**b**) rGONS-T.

The photoactive species involved in the reaction were investigated at natural pH (6.0) using EDTA, FFA, and t-BuOH, as scavengers for holes, singlet oxygen (^1^O_2_), and radicals, respectively. Figure 7a,b displays the OG concentration during the photocatalytic experiments with the addition of the scavengers for the GO-T and rGONS-T composites, respectively. The results indicate that for both photocatalysts the presence of any scavenger produces a decrease in the OG degradation rate, suggesting that photogenerated holes, ^1^O_2_, and radical species participate in the OG degradation pathway. 

For GO-T (Figure 7a), the addition of FFA and t-BuOH reduced the *k_app_* from 109.2 × 10^−3^ to 24.1 × 10^−3^ min^−1^ and from 109.2 × 10^−3^ to 16.6 × 10^−3^ min^−1^, respectively. Nevertheless, the highest reduction of the constant rate was found in the presence of EDTA (*k_app_* = 4.5 × 10^−3^ min^−1^). Regardless, the rGONS-T material, an analogous performance was observed (Figure 7b) and although the presence of t-BuOH and FFA led to a reduction of the photocatalytic activity, the addition of EDTA produced the highest decrease in the rate constant (from 48.4 × 10^−3^ to 21.3 × 10^−3^, 12.1 × 10^−3^ and 3.4 × 10^−3^ min^−1^, respectively). These results suggest that the photoactive species including hydroxyl, superoxide anion, hydroperoxyl radicals, and singlet oxygen (i.e., HO^●^, O_2_^●−^, HOO, and ^1^O_2_, respectively) participate in the photodegradation mechanism [66,67]. However, the high quenching effect of EDTA indicates that photogenerated holes (h^+^) play an essential role in OG removal for both GO-T and rGONS-T photocatalysts under simulated solar light. 

#### 3.2.3. Reutilization Tests

**Figure 8:**
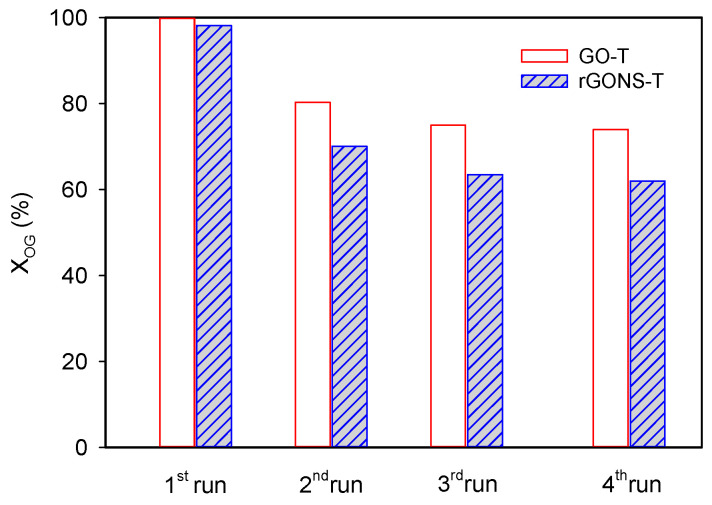
Reusability of the GO-T and rGONS-T photocatalysts for the OG degradation during four consecutive runs.

The photocatalytic stability of both GO-T and rGONS-T materials was examined. Four consecutive reusability cycles were performed at pH natural (pH = 6.0) under simulated solar light (Figure 8). The experiments were carried out as follows. After each run, the photocatalyst was filtered, washed with distilled water, and dried at 80 °C for 6 h. The resulting material was reused in the photocatalytic experiments using a fresh OG solution. The OG conversion decreased between the first and the second run for both photocatalysts (from 100% to 80.3% and from 98.2% to 70.1% for GO-T and rGONS-T, respectively), due to the adsorption of by-products on the photocatalyst. In the third run, the photocatalytic activity of the materials is essentially kept with a slight decrease of the OG conversion (i.e., from 80.3% to 75.0% and from 70.1% to 63.5% for GO-T and rGONS-T, respectively) and in the fourth run the photocatalytic performance of photocatalysts remained almost unchanged with respect to the third run concluding that both materials were stable under the reaction conditions used. Nevertheless, more studies will be required namely, the use in continuous flow reactors for the sake of checking the long-term stability of catalysts.

For comparison purposes, Table 3 comprises studies regarding graphene-TiO_2_ based photocatalysts that have been recently published towards dye degradation under visible light illumination. It can be concluded that the obtained photocatalyst in this work showed a good photocatalytic performance comparatively to that of other materials reported in the literature. 

## 4. Conclusions

The morphology of the graphene-TiO_2_ composite depends on the type of graphene derivative used during the synthesis. Thus, N/S- or B-doped graphene composites (rGONS-T and rGO-B) and undoped rGO composite (i.e., rGO-T) show clusters of TiO_2_ particles, while the composite including GO (GO-T) presents a structure formed by graphene layers coated by TiO_2_ particles. All materials were mesoporous and presented an acidic character. The addition of any graphene derivative triggers the narrowing band-gap energy in comparison with bare TiO_2_. 

The OG conversion and mineralization were always higher with the graphene derivative-TiO_2_ composites rather than TiO_2_ under simulated solar light.

The most active material consisted of GO in a TiO_2_ matrix (GO-T). The photocatalytic performance is being related with the morphology of GO-T composite that shows a strong interaction between graphene sheets and TiO_2_ particles, producing an optimal assembly of TiO_2_ on GO sheets as well as to the lowest band gap energy. 

The photocatalytic performance of functionalized graphene derivatives-TiO_2_ composites decreases as the initial solution pH increases, attaining a OG conversion of 100% and 99.5% for GO-T and rGONS-T, respectively at pH 3.0 due mainly to the occurrence of electrostatic attraction forces between the catalyst surface and OG molecules.

The addition of scavengers suggests that for both GO-T and rGONS-T catalysts, radical species, photogenerated holes, and singlet oxygen species participate in the OG degradation pathway. However, the higher reduction of the photocatalytic performance observed when EDTA is used as hole scavengers, indicates that photo-generated holes dominated the OG degradation on graphene-TiO_2_ composites rather than radicals or singlet oxygen species. In addition, the reutilization cycles prove that both GO-T and rGONS-T composites were stable in a series of consecutive runs.

## Figures and Tables

**Table 1:** Brunauer–Emmett–Teller (BET) surface area (*S*_BET_), total pore volume (*V*_pore_), mean pore diameter (*d*_pore_), pH at the point of zero charge (pH_PZC_), band-gap energy (E_g_), and crystallite size of synthetized materials.

Samples	*S*_BET_ (m^2^ g^−1^)	*V*_pore_ (cm^3^ g^−1^)	*d*_pore_ (nm)	pH_PZC_	E_g_ (eV)	Crystallite Size (nm)
TiO_2_	43	0.12	5.2, 10.3	3.5	3.20	9.7 ± 1.3
GO-T	55	0.17	4.2, 16.7, 25.2	3.1	2.98	12.1 ± 1.2
rGO-T	50	0.13	5.2, 10.3	3.2	3.15	--
rGONS-T	40	0.08	5.0, 7.1	3.3	3.12	7.6 ± 1.3
rGOB-T	80	0.20	5.0	3.2	3.12	8.9 ± 1.2

**Table 2:** OG conversion after 60 min (*X_OG_*), pseudo-first order kinetic rate constant (*k_app_*), and regression coefficient (*r*^2^) of OG.

Sample	*pH*	*X_OG_* (%)	*k_app_* (10^−3^ min^−1^)	r^2^
Photolysis	6.0	2.8	−−	−−
TiO_2_	6.0	47.6	11.2 ± 0.3	0.996
GO-T	6.0	99.8	109.2 ± 4	0.996
rGO-T	6.0	90.0	29.0 ± 3	0.97
rGONS-T	6.0	98.2	48.4 ± 2	0.993
rGOB-T	6.0	96.5	39.1 ± 2	0.99
GO-T	**3.0**	100.0	153.4 ± 8	0.992
GO-T	**10.0**	99.6	55.1 ± 3	0.98
GO-T + EDTA	6.0	22.1	4.5 ± 0.4	0.95
GO-T + *t*-BuOH	6.0	75.4	24.1 ± 1	0.996
GO-T + FFA	6.0	66.3	16.6 ± 1	0.98
rGONS-T	**3.0**	99.5	62.0 ± 3	0.992
rGONS-T	**10.0**	87.1	39.1 ± 1	0.994
rGONS-T + EDTA	6.0	16.4	3.4 ± 0.3	0.91
rGONS-T + *t*-BuOH	6.0	71.1	21.3 ± 0.3	0.999
rGONS-T + FFA	6.0	46.9	12.1 ± 0.8	0.95

**Table 3:** Compilation of recently published works regarding graphene-TiO_2_ based photocatalysts for dye degradation under visible light illumination.

Photocatalyst	Application	Main Results	Ref.
N-TiO_2_/rGO aerogel	20 mg L^−1^, 100 mL MB	Excellent adsorption Removal rates > 90% in 60 min	[68]
N-TiO_2_/rGO	10 mg L^−1^, 50 mL RhB	Degradation of 90% in 120 min	[69]
GO/TiO_2_/Hermin	10 mg L^−1^, 250 mL RhB under UV/Vis/H_2_O_2._	Degradation of 100% in 40 min	[70]
rGO/TiO_2_/WO_3_	MB	Degradation of 83% in 60 min	[71]
rGO/TiO_2_	5 mg L^−1^, 35 mL MB	Degradation of 82% in 140 min Conversion (TOC) 49% in 100 min	[72]
rGO/amino-grafted TiO_2_	5 mg L^−1^, MB 5 mg L^−1^, Rh6G	Degradation of 91.2% in 40 min (MB) Degradation of 88.3% in 240 min (Rh6G)	[73]
Graphene aerogel/TiO_2_/g-C_3_N_4_	20 mg L^−1^, 25 mL RhB	Adsorption of 96.5% in 60 min Degradation of 98.4% in 60 min	[74]
GO-TiO_2_	20 mg L^−1^, 50 mL OG	Degradation of 99.8% in 60 min Conversion (TOC) 40% in 60 min	This work
rGONS-TiO_2_	20 mg L^−1^, 50 mL OG	Degradation of 98.2% in 60 min Conversion (TOC) 22% in 60 min	This work
rGOB-TiO_2_	20 mg L^−1^, 50 mL OG	Degradation of 96.5% in 60 min Conversion (TOC) 17% in 60 min	This work

MB: Mthylene blue; RhB: Rhodamine B; Rh6G: Rhodamine 6G; OG: Acid Orange G.

## Supplementary Materials

The following are available online at https://www.mdpi.com/2079-4991/10/6/1106/s1, Figure S1: Normalized concentration of OG as a function of time pH values of 3.0, 6.0 (natural pH), and 10.0 for (a) GO-T and (b) rGONS-T.
