# Supplementary material for: Functionalized Graphene Derivatives and TiO2 for High Visible Light Photodegradation of Azo Dyes
Source: Nanomaterials (Basel). 2020 Jun 3;10(6):1106. doi: 10.3390/nano10061106 (PMC7353273; doi:10.3390/nano10061106)
Supplement: Supplementary file 1 [file nanomaterials-10-01106-s001.pdf]

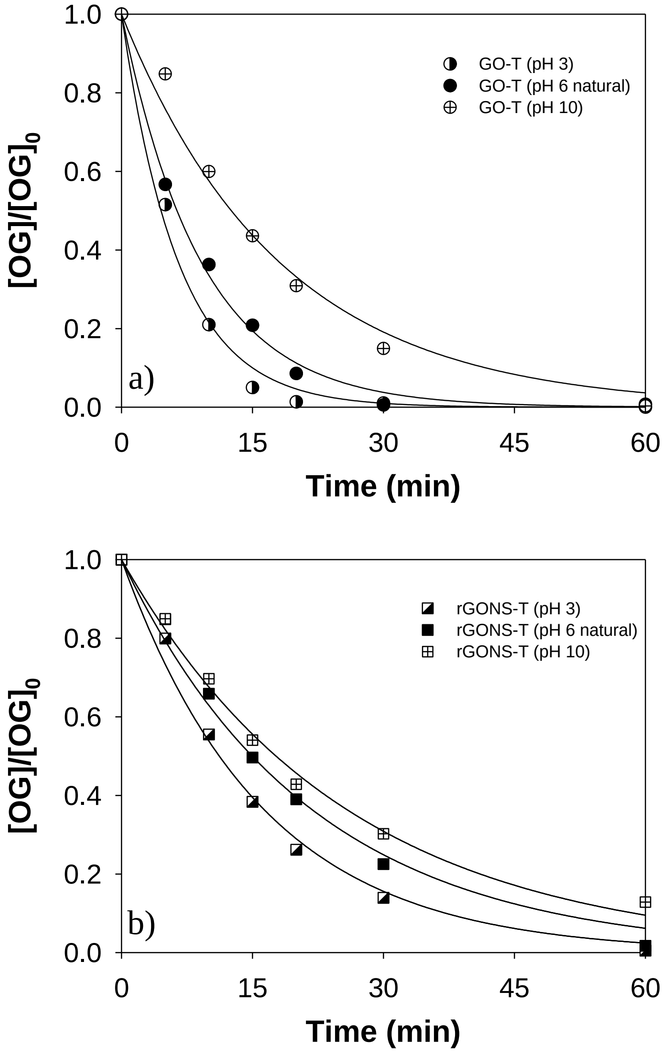

Figure S1. Normalized concentration of OG as a function of time pH values of 3.0, 6.0 (natural pH) and 10.0 for (a) GO-T and (b) rGONS-T.
